# Deep Semi Supervised Generative Learning for Automated Tumor Proportion Scoring on NSCLC Tissue Needle Biopsies

**DOI:** 10.1038/s41598-018-35501-5

**Published:** 2018-11-26

**Authors:** Ansh Kapil, Armin Meier, Aleksandra Zuraw, Keith E. Steele, Marlon C. Rebelatto, Günter Schmidt, Nicolas Brieu

**Affiliations:** 1 Definiens AG, Munich, 80636 Germany; 2 MedImmune LLC, Gaithersburg, MD 20878 USA

## Abstract

The level of PD-L1 expression in immunohistochemistry (IHC) assays is a key biomarker for the identification of Non-Small-Cell-Lung-Cancer (NSCLC) patients that may respond to anti PD-1/PD-L1 treatments. The quantification of PD-L1 expression currently includes the visual estimation by a pathologist of the percentage (tumor proportional scoring or TPS) of tumor cells showing PD-L1 staining. Known challenges like differences in positivity estimation around clinically relevant cut-offs and sub-optimal quality of samples makes visual scoring tedious and subjective, yielding a scoring variability between pathologists. In this work, we propose a novel deep learning solution that enables the first automated and objective scoring of PD-L1 expression in late stage NSCLC needle biopsies. To account for the low amount of tissue available in biopsy images and to restrict the amount of manual annotations necessary for training, we explore the use of semi-supervised approaches against standard fully supervised methods. We consolidate the manual annotations used for training as well the visual TPS scores used for quantitative evaluation with multiple pathologists. Concordance measures computed on a set of slides unseen during training provide evidence that our automatic scoring method matches visual scoring on the considered dataset while ensuring repeatability and objectivity.

## Introduction

The programmed death receptor 1 (PD-1) checkpoint protein with its ligand - programmed death ligand 1 (PD-L1) plays a major role in the immune escape of the cancerous tumor cells, i.e. in the inhibition of the human immune system responses^1,2^. More precisely, the proliferation and activation of T-cells as well as the production of the cytokine signaling proteins are inhibited by the binding of PD-L1 proteins to (i) the PD-1 receptors of activated T-cells and (ii) to the CD80/B7-1 receptors on T-cells and antigen presenting cell. Immunotherapeutic drugs aim at restoring the ability of immune cells to kill tumor cells by blocking this escape pathway. The role of complementary or companion diagnostics assays is, in this context, to help the identification of patients which are likely to benefit from a checkpoint inhibitor therapy, i.e. patients with high tumor PD-L1 levels^3^. The tumor PD-L1 level is estimated by a trained pathologist on small biopsy specimen stained with a PD-L1 antibody and most usually obtained in clinical practice with small needles. More precisely, its quantification is based on the tumor proportional score or TPS, which is defined as the percentage of tumor cells with PD-L1 staining localized in the membrane. For three of the four assays used for PD-L1 therapy of non-small cell lung cancer (NSCLC) and as detailed below, the negative or positive PD-L1 status of the patient is set by comparing this percentage to an assay specific cut-off value^4^.

There exists multiple assay systems to inform on PD-L1 treatment decision, each system consisting of an therapeutic agent (nivolumab, pembrolizumab, atezolizumab and durvalumab), a primary antibody clone (28-8 and 22C3 clones by Dako and SP142 and SP263 clones by Ventana respectively) and a reference standard cut-off value for setting the PD-L1 patient status^5–7^. For the 28-8 and 22C3 assays, PD-L1 status is defined based on the 50% standard cut-off on the TPS, the cut-off for the 22C3 assay being additionally extended to the 1% cut-off^7–9^. For the Ventana SP142 assay, PD-L1 status is defined based on the 50% cut-off on the TPS and on the 10% cut-off on the tumor area occupied by PD-L1 expressing infiltrating immune cells. Finally, for the Ventana SP263 assay, PD-L1 status is defined based on the 25% cut-off on the TPS. The reference scoring guidelines and more specifically the standard cut-off values for each assay system have been individually set and validated for their respective treatments in clinical trials to maximize correlation with patient outcome data^9,10^. It is interesting that some studies have shown the relative similarity of the two Dako and the Ventana SP263 assays^5,11,12^, providing some evidence that the three assays could be interchangeable. However, interchanging the reference standard cut-off values or defining a unique cut-off between assays could so far not be clinically demonstrated. Also, marked differences are reported while classifying patients as positive or negative if the same cut-off is enforced between the assays^4,7^. This seems to have been recently confirmed by Munari *et al*.^13^ which report that the proportion of positive cases at the cut-off values associated with the Dako 22C3 clone significantly differs if the slides are stained with the Ventana SP263 clone and not with the Dako 22C3 clone as intended. In this work, we solely consider the Ventana SP263 PD-L1 system which was developed with durvalumab. As detailed in the corresponding development study^10^, the cut-off value at 25% was set to maximize the predictive value of the test on durvalumab treated patients while considering other parameters such as the prevalence of the population, the ease of visual scoring, and optimizing for the negative predictive value. Because the focus of this work is on the SP263 assay system, that other cut-point values have not been shown to be clinically relevant for this assay, and that the interchangeability between different assays is still an open topic of research, we consider only the standard reference 25% cut-off in the remaining of this study.

There are known challenges to an accurate estimation of TPS^14^. First, PD-L1 staining is not restricted to the membrane of tumor cells: tumor cells with strong cytoplasmic but no membrane staining, immune cells (e.g. macrophage and lymphocyte) as well as necrotic and stromal regions are not included in the score calculation despite possibly showing PD-L1 staining. A challenge specific to visual scoring is moreover the difficulty for any human observer to estimate heterogeneous distribution of cell populations, with positive and negative tumor regions being often spatially inter-mixed. These challenges make TPS estimation a subject to some variability among pathologists^12^. In this work, we propose an automatic scoring solution based on image analysis which achieves an accuracy similar to visual scoring while ensuring objective and reproducible scores and could potentially be used as a computer aided system to help pathologists make a better therapeutic decision. The complexity of the scene and the difficulty of the task naturally lead us to opt for a deep learning-based solution.

Previous works have shown the ability of deep learning-based methods to solve complex tasks in the field of image analysis and understanding^15–20^ as well as in the more specific field of digital pathology image analysis^21–24^. As a first example, Litjens *et al*. showed in their pioneering study^21^ the potential of deep learning for the detection of prostate cancer regions and of breast cancer metastasis in lymph nodes on digital images of H&E stained slides. More specially, two fully supervised convolutional neural networks (CNN)^25,26^ were independently trained on the complete manual annotations of 200 and 170 tissue slides respectively. Cruz-Roa *et al*. proposed a similar fully supervised CNN-based approach for the detection of invasive breast cancer region in H&E stained slides^22^, relying on the annotations of nearly 400 slides from multiple different sites for training. Most previous works in the field of digital pathology image analysis build on fully supervised networks, which are trained only on labeled information obtained through very extensive manual annotations. Collecting the necessary amount of annotations is however a well known problem in this field. This is because images with the level of complexity observed in digital pathology can and should only be interpreted by experts with several years of training and experience. This is a key difference to other fields of application of deep learning methods, for instance in robotics, for which the complexity arises more from the number and diversity of the classes than on the ability of untrained humans to recognize these classes. Because pathologists can disagree on the interpretation of a given slide, it is often necessary to collect manual annotations of the same slide from different pathologists. While being often necessary in order to reduce ambiguity in the training set, annotating the same slide multiple times significantly increases the burden of manual annotation.

To bypass the need of manual annotation for region segmentation or object detection, some recent works proposed to directly infer the patient label. Bychkov *et al*.^27^ introduced a long short term memory (LSTM) network to directly predict patient outcome on tissue microarrays. Campanella *et al*.^28^ developed a multiple instance learning (MIL) solution to predict prostate cancer diagnosis on needle biopsies, the training of the system requiring a huge dataset of more than 12000 slides. The use of needle biopsies and the small size of datasets available in clinical trial studies make however the use of the aforementioned weakly supervised learning approaches challenging in our scenario.

As previously shown by Vandenberghe *et al*.^29^ for the Her2 scoring of breast cancer slides, scores do not have to be learned and can be accurately replicated using the heuristic definition given in the scoring guidelines and an intermediate detection step. To keep the intermediate detection step while reducing the amount of manual annotation, we propose a semi-supervised learning solution. These approaches still employ manual annotations but make use of raw unlabeled data to lower the necessary amount of labeled data^30^. Only a few previous works^31–33^ have used semi-supervised learning for digital pathology image analysis. Peikari *et al*. recently introduced a cluster-then-label method based on support vector machine classifier that is shown to outperform classical supervised classifiers^31^. Sparks *et al*. proposed an image query approach based on semi-supervised manifold learning^34^. Other works focus on transfer learning where the model weights are initialized on other classification task^32,33^ or learned on raw unlabeled data using representation learning, the labeled data being then used for model refinement only. Given the recent advances in the field of generative adversarial networks (GAN), our work build on class auxiliary generative adversarial networks (AC-GANs). To the best of our knowledge, this study introduces the first application of AC-GAN networks for digital pathology image analysis as well as the first computer-aided diagnostic tool for PD-L1 scoring on needle biopsies.

## Methods

The proposed algorithm for the automated TPS estimation consists of two main steps. First, positive tumor cell regions TC(+) and negative tumor cell regions TC(−) are detected using a deep semi-supervised architecture trained on both labeled and unlabeled data. An Auxiliary Classifier Generative Adversarial Network (AC-GAN)^35^ is more precisely chosen to this end. Second, TPS is computed as the ratio between the pixel count (i.e. the area) of the detected positive tumor cell regions to the pixel count of all detected tumor cell regions. Since approaches based on pixel counts often show higher performance than cell-count based quantification^36^ and enable an easier annotation workflow, we estimate TPS based on the pixel counts of PD-L1 positive and negative tumor regions.

### Dataset consolidation for region detection

A small subset of slides is selected and partially annotated by two pathologists for positive tumor cells TC(+), negative tumor cells TC(−), positive lymphocytes, negative lymphocytes, macrophages, necrosis and stromal regions. A simple detection of the tissue and background regions based on Otsu thresholding^37^ and morphological filtering is performed, leading to the introduction of another class corresponding to non-tissue regions. Labeled image patches are generated on a regular grid defined on the annotated regions that are concordant between the two pathologists. This leads regions with different classes to be discarded from the analysis. On the remaining set of non-annotated slides, unlabeled patches are generated on a regular grid defined on the detected tissue.

### Auxiliary classifier generative adversarial networks (AC-GAN)

Let’s first recall the principle and architecture of Generative Adversarial Networks introduced by Goodfellow *et al*.^35^. GANs consist of two neural networks, a generator network (G) and a discriminator network (D). The network G transforms a noise vector *z*, which is sampled from a simple distribution such as an uniform or normal distribution, into an fake image *X*_*fake*_ = *G*(*z*) using a series of deconvolution and activation layers. The network D classifies an input image as real or fake. More formally, the discriminator outputs the probability distribution over the sources *P*(*S*|*X*), *S* ∈ {*real*, *fake*}, through a series of convolutional and activation layers by maximizing the log-likelihood of the correct source:

1 $${L}_{S}={\mathbb{E}}[log\,P(S=real|{X}_{real})]+{\mathbb{E}}[log\,P(S=fake|{X}_{fake})]$$

The two networks are trained in opposition following a minimax game^38^ formally defined as follows:

2 $$\mathop{{\rm{\min }}}\limits_{G}\,\mathop{{\rm{\max }}}\limits_{D}\,V(D,G)={{\mathbb{E}}}_{X\sim {P}_{data}}[\mathrm{log}\,D(X)]+{{\mathbb{E}}}_{z\sim noise}[\mathrm{log}\,(1-D(G(z)))]$$

More intuitively, the discriminator is trained to differentiate whether the image is coming from real image distribution or fake image distribution from G. In the opposition, G is trained to produce images which are more and more difficult to be identified by the discriminator as real or fake images. When the optimum of this minimax game is reached, the generator creates images so close to the real images that they cannot be differentiated by the discriminator.

The GAN architecture is, however, strictly unsupervised and generative in nature i.e. it can only be used to generate realistic new samples, leading its current main application in digital pathology to be stain normalization^39,40^. Some recent works^41,42^ in the computer vision community proposed its extension to the AC-GAN variant. The AC-GAN leverages the side information on the class labels by two means. First, the generator is conditioned with class labels to perform conditional image generation: the generator network takes as input a noise vector concatenated with the one-hot embedded class labels. The concatenated vector goes through a series of transformations by a CNN to create fake images *X*_*fake*_ = *G*(*c*, *z*). Second, the discriminator, in addition to predicting the correct source probability *P*(*S*|*X*), also performs class label recovery. The discriminator contains an auxiliary classifier network which outputs the probability distribution of the class *P*(*C*|*X*). The network parameters between the auxiliary classifier and the discriminator are shared, which enables joint learning of *P*(*C*|*X*) and *P*(*S*|*X*). This requires the introduction of a second cost function as the log-likelihood of the correct class:

3 $${L}_{C}={\mathbb{E}}[log\,P(C=c|{X}_{real})]+{\mathbb{E}}[log\,P(C=c|{X}_{fake})].$$

**Figure 1:**
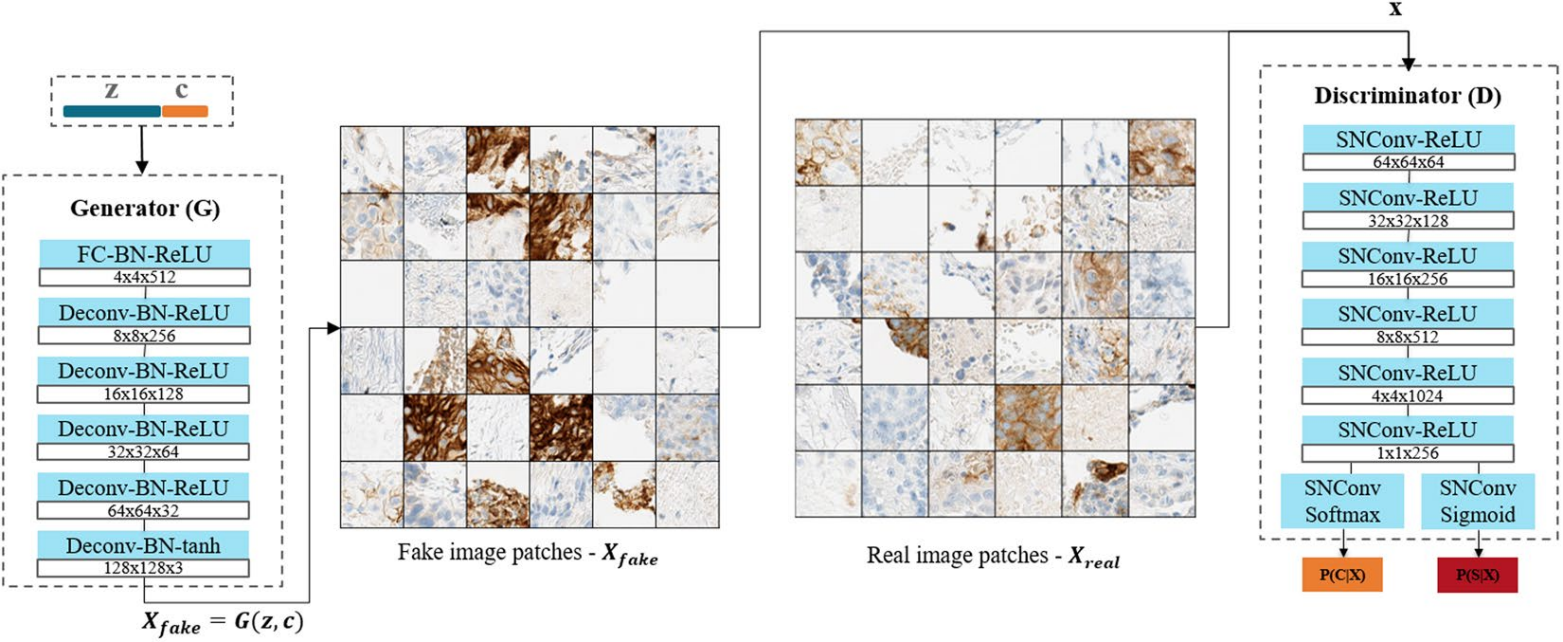
Proposed AC-GAN architecture. The noise *z* and the class one-hot encoding *c* are concatenated and sent to the generator (G). The generator creates class-conditioned fake images. The classes *c* and the source information *s* are jointly predicted by the discriminator (D) given a fake (left) or a real (right) input. *SNConv* implies convolutional layers using spectral normalization^51^, which introduces a minor modification to the original AC-GAN architecture^41^.

Similar to the vanilla GAN formulation, the two networks D and G are trained by a minimax game with slight modification. The discriminator D is trained to maximize *L*_*C*_ + *L*_*S*_ and the generator G to minimize *L*_*S*_ − *L*_*C*_. Since the maximization and minimization problems are interlaced, the AC-GAN model is trained by alternatively updating the two models G and D. The exact AC-GAN architecture used in this work is displayed in Fig. 1.

In our application, we are only interested in the classification performance of the network. While we investigate the discriminator performance in more detail, further quantitative assessment of generator has not been performed. Some qualitative examples of images produced by the generator are however displayed in Fig. 1.

### Fully-supervised, non-generative and generative semi-supervised networks

We test the semi-supervised generative AC-GAN architecture against two baseline classification networks for fully-supervised learning and two baseline non-generative networks for semi-supervised learning. The two chosen fully-supervised architectures, the inception network v2^43^ and a shallow VGG^25^ network modified to be fully-convolutional and to include batch-normalization^44^ are commonly employed for the analysis of digital pathology images. The two non-generative semi-supervised networks are vanilla auto-encoder networks^45^ built on the two aforementioned classification networks

## Experiments and Results

### Manual annotation and visual TPS datasets

Our dataset consists of 270 NSCLC needle biopsy slides from a subset of the clinical trials (NCT01693562) and (NCT02000947). The slides are stained with the Ventana PD-L1 (SP263) assay^10^ and scanned on an Aperio scanner. Scene resolution is 0.49 *μm* per pixel. On a subset of *N*_*score*_ = 60 slides, visual TPSs are estimated on the scanned digital slides by two in-house pathologists. This completes the visual TPS estimated on glass-slides and obtained from an external source (Ventana Medical System Inc.). A smaller subset *N*_*cnm*_ = 20 is selected across the range of TPS values for training and testing the supervised part of the region detection model and have as such been partially manually annotated by the two in-house pathologists using an in-house annotation software. More specifically, 15 slides are used for training and 5 slides for testing and optimizing the model parameters. The accuracy of the resulting computer-based TPS is estimated against the three visual TPS values on the remaining *N*_*test*_ = 40 unseen slides, i.e. unused for training or testing the supervised nor the unsupervised part of the region detection model.

### Model training

Patches of size 128 × 128 pixels are extracted on a regular grid of 20 pixel stride defined on the concordant annotated regions and are augmented using 90 degree rotation. We sample unlabeled patches on a regular grid of 60 pixel stride defined on the tissue area of the 210 slides which have not been scored by all pathologists as well as on the 15 annotated slides which are used for generating the labeled training database. This yields a total of around 180k labeled and 400k unlabeled patches for training as well as 40 k labeled patches for testing. All models are trained using the same patches. Batches with 64 labeled patches are used for training the fully-supervised networks. Batches with 32 labeled patches and 32 unlabeled patches are used for training the three semi-supervised networks. For the two non-generative semi-supervised networks, the reconstruction loss is computed on the complete batch and the classification loss on the labeled patches only. For the AC-GAN, the generative loss is computed on the complete batch and the classification loss on the labeled patches only. Training of the four baseline networks and of the generator and discriminator of the AC-GAN network is performed for 100 *k* and 200 *k* iterations respectively using the Adam optimizer^46^ with the following learning parameters: *lr* = 0.0001, *beta*1 = 0.5, *beta*2 = 0.999. For each network, we select the model weights that maximize the accuracy on the test dataset in order to avoid overfitting on the training set. The developed framework is based on the open-source Keras API^47^ and Tensorflow framework^48^.

### Prediction and automated TPS estimation

**Figure 2:**
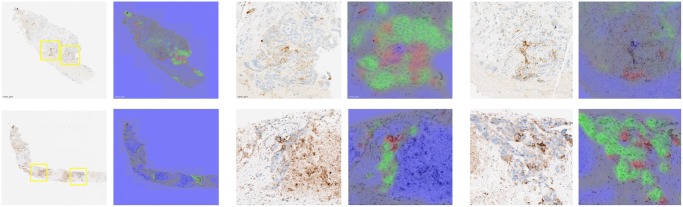
Example of class probability maps obtained with AC-GAN model. Left: Original images. Right: Predicted probabilities associated to positive tumor cell region TC(+) (red channel), negative tumor cell region (green channel) and to other classes (blue channel) superimposed to the original grayscale layer. The two yellow boxes overlaid to the far left images correspond to the regions of interest displayed on the right.

The prediction is restricted to the detected tissue regions and is performed in a sliding window manner with a stride of 32 pixels. The ability of the system to differentiate between the classes has been qualitatively checked on the *N*_*test*_ unseen slides. An example of region detection results is provided in Fig. 2. Quantitatively, on our reduced dataset of manually annotated regions, the percentage of pixels in PD-L1 positive tumor cell region that are wrongly classified as being in a macrophage region is less than 7%, the percentage of pixels in macrophage region that are classified as being in a PD-L1 positive tumor cell region is less than 14%, specifically showing the ability of the proposed system to differentiate between different types of PD-L1 stained cell regions. On each of the *N*_*score*_ = 60 slides for which three visual TPS values are available, we predict the different class probabilities and assign each pixel to the class label of the maximum probability. Given the resulting TC(+) and TC(−) pixels in a given slide, we approximate the corresponding TPS as the ratio of the number of tumor positive cell pixels to the total number of tumor cell pixels:

4 $$TP{S}_{cnn}=\frac{\#TC(\,+\,)}{\#TC(\,-\,)+\#TC(\,+\,)}\mathrm{.}$$

**Figure 3:**
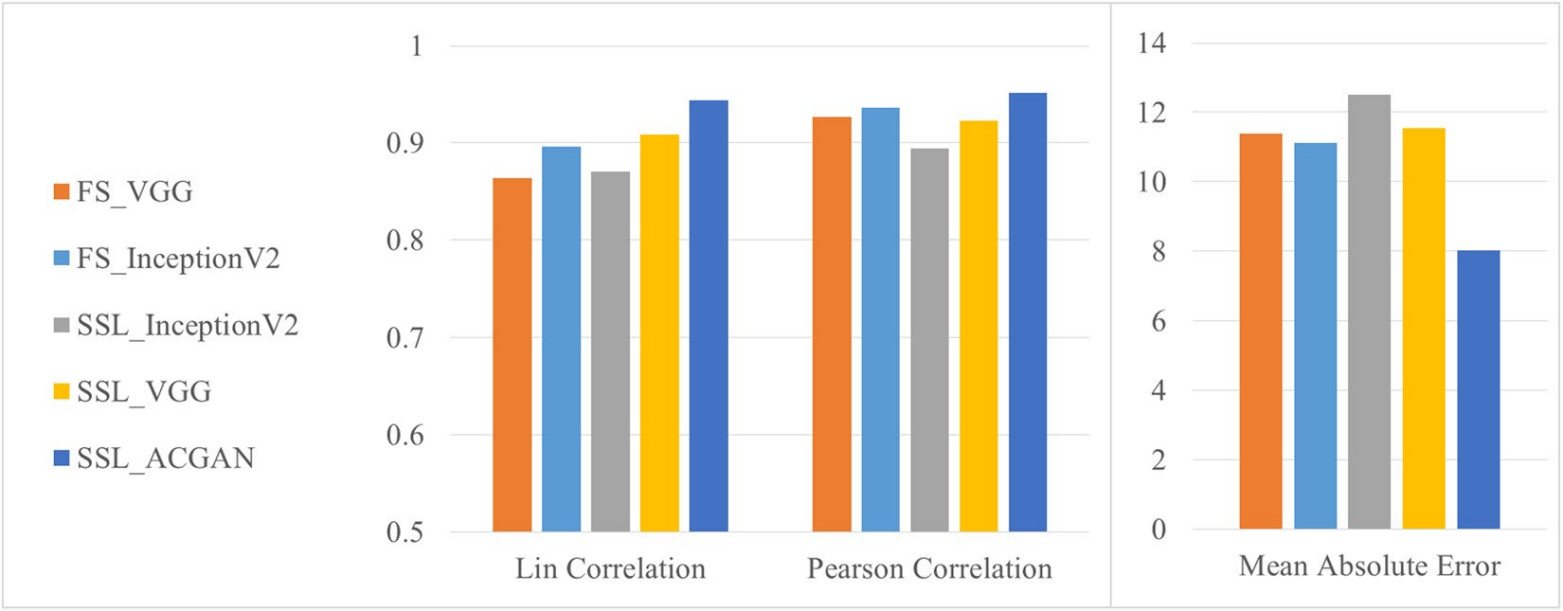
Concordance measures between the consolidated visual TPS and the automated computer-based TPS. The automated TPS is calculated from the tumor regions detected using different supervised and semi-supervised network architectures: fully-supervised shallow VGG net (FS-VGG), fully-supervised inception net v2 (FS-InceptionV2), semi-supervised VGG net (SSL-VGG), semi-supervised inception net v2 (SSL-InceptionV2), and semi-supervised AC-GAN (SLL-ACGAN). Values are computed on the *N*_*test*_ cases unseen during training or testing of the networks.

We then compute for each slide the consolidated visual score *TPS*_*ref*._ as the median of the three visual scores. The following concordance measures between the automated computer-based TPS and the consolidated visual score is calculated on the set of 40 unseen slides only to ensure an independent estimation of the performance: Lin’s concordance coefficient (Lcc), Pearson correlation coefficient (Pcc) and Mean Absolute Error (MAE). As reported in Fig. 3, the AC-GAN achieves on all measures a higher level of agreement to the visual scores (*Lcc* = 0.94, *Pcc* = 0.95, *MSE* = 8.03) than any other fully-supervised and semi-supervised network.

### Inter-rater variability of visual TPSs

**Figure 4:**
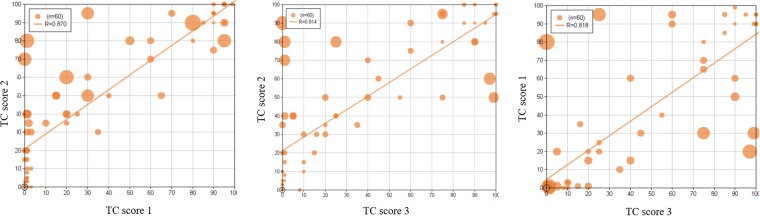
Pairwise scatter plot between the three reference visual TPSs. Each disk represents one of the 60 visually scored slides. For a given case, the size of the disk is proportional to the inter-rater variability Δ_*path*_.

To quantify the variability between the visual scores (cf. Fig. 4), we additionally estimate for each slide the inter-rater variability Δ_*path*._ as the mean absolute pairwise difference between the associated visual scores:

5 $${{\rm{\Delta }}}_{path\mathrm{.}}=\frac{1}{6}\sum _{1\le i\le 3i < j\le 3}\,|TP{S}_{pat{h}_{i}}-TP{S}_{pat{h}_{j}}|.$$

**Figure 5:**
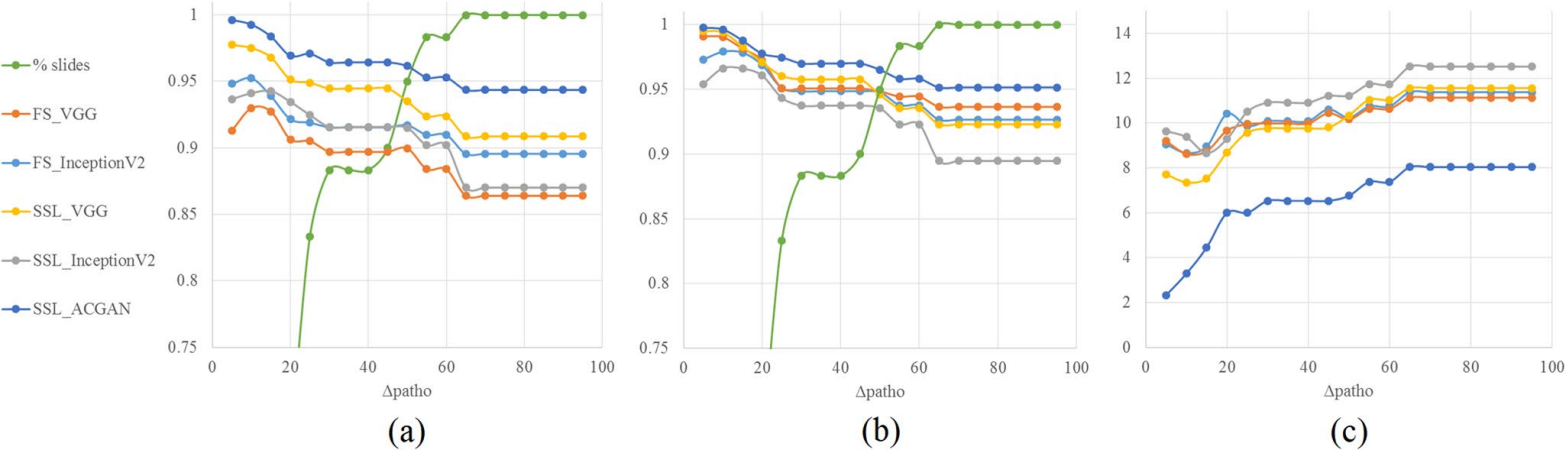
Concordance measures between the consolidated visual TPS and the computer-based TPS estimated from the tumor regions detected using the different supervised and semi-supervised network architectures, for increasing maximum levels of inter-rater variability Δ_*patho.*_ on the x-axis. (**a**) Lin’s concordance coefficient, (**b**) Pearson Correlation Coefficient and (**c**) Mean Absolute Error. The green curve indicates the percentage of slides for which the inter-rater variability stays below the given level.

Please note that Tsao *et al*. recently confirmed the high concordance of PD-L1 scoring on glass slides and digital slides^12^, leading us to pool of all data to estimate inter-rater variability independently of a glass/digital scoring. Given increasing maximum levels of inter-rater variability, we restrict the computation of the aforementioned concordance measures to the slides whose associated value stays below the given maximum. As displayed in Fig. 5, the automated TPS become more concordant to the consolidated visual scores the more concordant the visual scores are. A Lin’s concordance coefficient of 0.96 is for example reached on the TPSs obtained with the AC-GAN architecture on cases for which the inter-rater variability is smaller than 40%. The better performance of the semi-supervised generative AC-GAN network over the other networks is consistent across highly-concordant and low-concordant cases.

### Automated AC-GAN scoring and visual TPS estimation

We compare hereby in more detail the performance of the automated score based on the AC-GAN detection to the three visual scores by pathologists. Table 1 reports the same concordance measures as above between all the visual scores and the AC-GAN score. To study the ability of the proposed automated solution to determine the patient status, we additionally compute the Overall Percent Agreement (OPA), Negative Percent Agreement (NPA) and Positive Percent Agreement (PPA) at the 25% cut-off (cf. Table 2). As detailed in the introduction, this cut-off value is specific to the SP263 PD-L1 clone and has been shown to optimize the probability of responses to treatment^10^.

**Table 1:** Pairwise Lin’s concordance/Pearson correlation coefficients and Mean Absolute Error (Lcc/Pcc/MAE) between every visual TPS and the automated TPS estimation based on AC-GAN.

	*TPS*_1_			*TPS*_2_			*TPS*_3_			*TPS*_*cnm*_		
	Lcc	Pcc	MAE	Lcc	Pcc	MAE	Lcc	Pcc	MAE	Lcc	Pcc	MAE
*TPS*_1_	—	—	—	0.84*	0.90*	15.3	0.81	0.82	13.2	0.95*	0.95*	7.4*
*TPS*_2_	0.84	0.90	15.3	—	—	—	0.79	0.82	16.6	0.84	0.89	15.0
*TPS*_3_	0.81	0.82	13.2	0.79	0.82	16.6	—	—	—	0.88	0.89	11.3
*TPS*_*cnm*_	0.95*	0.95*	7.4*	0.84*	0.89	15.0*	0.88*	0.89*	11.3*	—	—	—

The star indicates for each concordance measure the TPS yielding the best performance, i.e. the maximum value for Lcc and Pcc and the minimum value for the MAE. Values are computed on the *N*_*test*_ cases unseen during training or testing of the AC-GAN model.

**Table 2:** Pairwise Overall, Negative and Positive Percent Agreement (OPA/NPA/PPA) at the 25% cut-off between every visual TPS and the automated TPS estimation based on AC-GAN.

	*TPS*_1_			*TPS*_2_			*TPS*_3_			*TPS*_*cnm*_		
	OPA	NPA	PPA	OPA	NPA	PPA	OPA	NPA	PPA	OPA	NPA	PPA
*TPS*_1_	—	—	—	0.78	0.62	1.00	0.88*	0.88	0.88*	0.90*	0.88	0.94*
*TPS*_2_	0.78	1.00*	0.65	—	—	—	0.80	1.00*	0.69	0.83	1.00*	0.73
*TPS*_3_	0.88	0.91	0.83	0.80	0.65	1.00	—	—	—	0.88	0.87	0.89
*TPS*_*cnm*_	0.90*	0.95	0.84*	0.83*	0.68*	1.00*	0.88*	0.91	0.84	—	—	—

The star indicates for each concordance measure the TPS yielding the best performance, i.e. the maximum value. Values are computed on the *N*_*test*_ cases unseen during training or testing of the AC-GAN model.

The automated TPS computed from the AC-GAN predicted regions yields the highest correlation and the lowest absolute error for all but one case. Note that in the only case where this does not hold, the Pearson correlation of the automated score *TPS*_*cnm*_ = 89 is very close to the highest value *TPS*_1_ = 0.90. Computing the concordance to the visual scores *TPS*_3_ estimated on the microscope, we note that the automated TPS scores leads to a higher agreement (Lcc = 0.88, Pcc = 0.89, MAE = 11.3) than the two pathologist scores estimated on digital slides (*TPS*_1_:Lcc = 0.81, Pcc = 0.82, MAE = 13.2) − (*TPS*_2_:Lcc = 0.79, Pcc = 0.82, MAE = 16.6). These observations are confirmed on the reported OPA values of the low/high PD-L1 status at 25% cut-off. The automated scoring systematically yields the highest agreement with the visual TPSs. In particular, considering the third visual TPS, automated scoring achieves an *OPA* of 0.88 to be compared with OPAs of 0.88 and 0.80 observed for the first and second pathologists visually scoring on digital slides.

**Figure 6:**
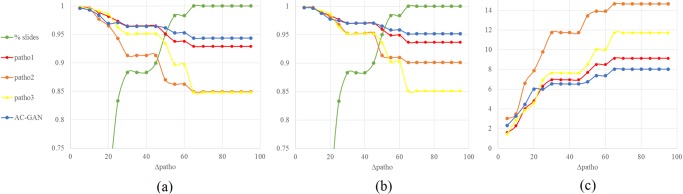
Considering the three visual TPSs and the TPS estimated using the region detected by the AC-GAN network, concordance measures between each TPS and the median of the three remaining TPSs for increasing maximum levels of inter-rater variability Δ_*patho*._ on the x-axis. (**a**) Lin’s concordance coefficient. (**b**) Pearson Correlation Coefficient and (**c**) Mean Absolute Error. The green curve indicates the percentage of slides for which the inter-rater variability stays below the given level.

**Figure 7:**
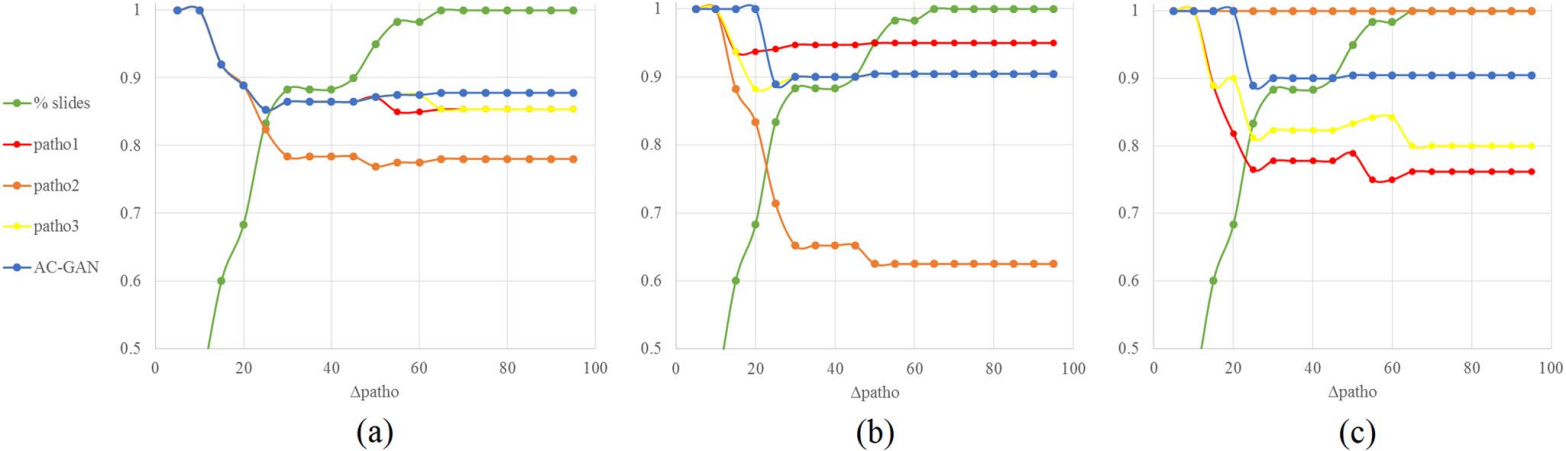
Considering the three visual TPSs and the TPS estimated using the region detected by the AC-GAN network, concordance measures between the patient status estimated from each TPS and patient status estimated from the median of the three remaining TPSs for increasing maximum levels of inter-rater variability Δ_*patho*._ on the x-axis. (**a**) Overall Percent Agreement. (**b**) Negative Percent Agreement and (c) Positive Percent Agreement. The green curve indicates the percentage of slides for which the inter-rater variability stays below the given level.

To further analyze the concordance of automated scoring versus visual scoring, we consider the AC-GAN score and the three visual scores independently of their source and compute for each of the four resulting scores all concordance measures between each score and the median of the three remaining scores. Results displayed in Table 3, Figs 6 and 7 provide further evidence of the good performance of the computer-based automated TPS estimation. In all measures, the automated score systematically outperforms the visual scores.

**Table 3:** Concordance measures between the visual and the automated TPSs against the median of the other scores on the *N*_*test*_ cases unseen during training or testing of the AC-GAN model.

	*TPS*_1_	*TPS*_2_	*TPS*_3_	*TPS*_*cnm*_
Lin/Pearson/MAE	0.93/0.94/9.14	0.85/0.90/14.65	0.85/0.85/11.73	0.94/0.95/8.00
OPA/NPA/PPA	0.85/0.95/0.76	0.78/0.63/1.0	0.85/0.90/0.80	0.88/0.90/0.85

## Discussion and Conclusion

The aim of anti-PDL1 therapies is to revive the immune response to cancer cells: inhibiting the PD-L1 pathway reverses T-cell exhaustion and restores T cell’s cytotoxic activity. Patients with high expression generally showing higher response rates and longer progression free survival than patients with low expression, an accurate testing of PD-L1 expression may inform on the best treatment decision on whether or not the patient should follow such therapy. As we recalled in the introduction, there is a significant heterogeneity between the existing PD-L1 assay system, different antibodies being employed as companion and complementary diagnostics for different immunotherapeutic drugs, different patterns of staining (tumor cells only or tumor cells and tumor infiltrating immune cells) being considered for different indications (NSCL resp. urothelial carcinoma) and finally different clinically relevant cut-off values being used for different antibodies for a given indication. While the focus of this work is to replicate the test associated to the antibody clone SP263 on NSCLC patients^10^, we present what is to our knowledge the first proof of concept study showing that deep learning enables an accurate and automated estimation of the PD-L1 expression level and PD-L1 status on small biopsies samples.

The performed analysis of inter-rater variability suggests that the accuracy achieved by the proposed automated scoring method is concordant with visual scoring by pathologists on our dataset. This work focuses on the automated estimation of the PD-L1 tumor proportion score yet, it more generally introduces the first application of deep semi-supervised and generative learning networks (AC-GAN) in the field of digital pathology. It also provides first evidence of the good performance of this model against standard fully supervised learning networks.

The proposed system takes 245 s per cm^2^ to detect the regions and to compute the score using a single NVidia K80 GPU chip. Given the slide resolution of 0.5 *μm*, this corresponds to predicting on an image of around 20 *K* × 20 *K* pixels. This translates into an average computation time of 78 s per biopsy. Because the AC-GAN architecture can be further optimized for speed upon transformation into a fully convolutional network^49^, that the prediction can be parallelized on multiple GPU chips and that the time to estimate the score from the detected regions is neglectable, the scoring system is foreseen to take only a few seconds in a potential diagnostic setup.

Going beyond the presented proof of concept, we believe that further evidence could be provided by increasing the size of the unseen dataset on which the agreement between the automated and visual TPSs has been computed as well as ensuring that the comparison between the visual scores is not biased by external parameters such as (i) the heterogeneous experience of the pathologists and (ii) if the scoring is performed on digital or glass slides. Also, the interoperability of the system should be further analyzed, for instance by applying the trained model on an independent patient cohort in particular on data generated by a different diagnostic laboratory. While we focus in this study on the technical aspects of the image analysis and automated scoring algorithm to include its technical performance compared to manual scoring by a pathologist, the use of the proposed algorithm as a predictive signature of response to durvalumab represents a logical extension of this work and is currently under study. Getting confirmation results will be key before potentially applying the proposed system into clinical routine.

Even though we currently do not have data to support this claim, we would expect that the proposed method could be applied to the 22C3 and 28-8 Dako PD-L1 assays provided that enough manual annotations are available for every class of interest in each assay. This is first because the estimation of the PD-L1 status solely depends on the TPS and second because these two assays appear relatively similar in overall pattern with the SP263 assay. However, because each PD-L1 clone is associated with a different clinically relevant cut-off value, the determination of the patient status would have be adapted according to their respective guidelines. Since staining of tumor cells with the Ventana SP142 assay has been shown to be less concordant (e.g. to stain fewer tumor cells) and that the decision on the PD-L1 status is not only based on TPS but also on the percentage of tumor area occupied by PD-L1 expressing tumor-infiltrating immune cells, we do not foresee that the proposed algorithm could be used at this point to determine patient status on this assay system. However, because the determination of the PD-L1 status in urothelial carcinoma (UC) tissue involves the scoring of both tumor cells and immune cell regions and that durvalumab has demonstrated favorable clinical activity in locally advanced/metastatic UC^50^, we do foresee a computer-based quantification of tumor-associated immune cells on the SP263 assay as a logical next step. In this case, an additional challenge to be solved is the low agreement among pathologists on immune scoring^12^. More generally, we envision that the success of checkpoint inhibitor related immune therapies can be increased by automated profiling of tumor and immune cells with respect to their cluster of differentiation (CD) protein expression levels. This study on CD274 (PD-L1) in NSCLC is the first conclusive step in this journey.

## Data Availability

The source code will be made available upon reasonable request to the corresponding author. The datasets generated during and/or analyzed during the current study are not publicly available due to ongoing work on the data analysis.
